# Impact of renin–angiotensin–aldosterone-system inhibitor drugs on mortality in patients with atrial fibrillation and hypertension

**DOI:** 10.1186/s12872-022-02580-2

**Published:** 2022-04-01

**Authors:** Wei Xu, Yan-min Yang, Jun Zhu, Shuang Wu, Juan Wang, Han Zhang, Xing-hui Shao

**Affiliations:** Emergency Center, Fuwai Hospital, National Center for Cardiovascular Diseases, Chinese Academy of Medical Sciences and Peking Union Medical College, No. 167 Beilishi Road, Xicheng District, Beijing, People’s Republic of China

**Keywords:** Atrial fibrillation, Hypertension, Angiotensin-converting enzyme inhibitors, Angiotensin receptor blockades, Cardiovascular disease, Renin–angiotensin–aldosterone-system, Mortality

## Abstract

**Background:**

Renin–angiotensin–aldosterone-system inhibitors markedly play an active role in the primary prevention of atrial fibrillation (AF), but the impact of angiotensin-converting enzyme inhibitors (ACEIs) or angiotensin receptor blockers (ARBs) on the mortality of patients with AF remains unclear. This study aimed to examine the relationship between treatment with ACEIs or ARBs and mortality in emergency department (ED) patients with AF and hypertension.

**Methods:**

This multicenter study enrolled 2016 ED patients from September 2008 to April 2011; 1110 patients with AF and hypertension were analyzed. Patients were grouped according to whether they were treated with ACEI/ARB or not and completed a 1-year follow-up to evaluate outcomes including all-cause death, cardiovascular death, stroke, and major adverse events (MAEs).

**Results:**

Among the 1110 patients with AF and hypertension, 574 (51.7%) received ACEI/ARB treatment. During the 1-year follow-up, 169 all-cause deaths (15.2%) and 100 cardiovascular deaths (9.0%) occurred, while 98 strokes (8.8%) and 255 MAEs (23.0%) occurred. According to the multivariate Cox regression analysis, ACEI/ARB therapy was significantly associated with a reduced risk of all-cause death (HR, 0.605; 95% CI 0.431–0.849; *P* = 0.004). Moreover, ACEI/ARB therapy was independently associated with a reduced risk of cardiovascular death (HR 0.585; 95% CI 0.372–0.921; *P* = 0.020) and MAEs (HR 0.651, 95% CI 0.496–0.855, *P* = 0.002) after adjusting for other risk factors.

**Conclusions:**

Our results revealed that ACEI/ARB therapy was independently associated with a reduced risk of all-cause death, cardiovascular death, and MAEs in ED patients with AF and hypertension. These results provide evidence for a tertiary preventive treatment for patients with AF and hypertension.

**Supplementary Information:**

The online version contains supplementary material available at 10.1186/s12872-022-02580-2.

## Introduction

Atrial fibrillation (AF) is a common arrhythmia encountered in the clinic, being associated with a rising risk of mortality or stroke and, therefore, is a global public health concern [1, 2]. As AF is a manifestation of hypertensive heart disease, patients with hypertension are prone to cardiac arrhythmias, especially AF [3]. The development of AF is characterized by electrical modifications and atrial structural remodeling [4–6]. Particularly, atrial structural remodeling is characterized by atrial enlargement and fibrosis [7, 8]. Various animal model studies of AF reiterated that the activation of the renin–angiotensin–aldosterone system (RAAS) leads to fibrosis and myocardial structural remodeling and demonstrated the cardioprotective effects of angiotensin-converting enzyme inhibitors (ACEIs) or angiotensin receptor blockers (ARBs) and anti-inflammatory drugs with antioxidant properties [9, 10]. It has been fully investigated in the effect of RAAS inhibitors on the primary and secondary prevention of AF [11, 12]. However, the impact of ACEI/ARB on the mortality of patients with AF remains unclear. Meanwhile, it is difficult to distinguish whether the benefit occurs from blood pressure (BP) control and its accompanying hemodynamic changes or because of RAAS inhibition. Thus, we performed a study on patients with AF and hypertension with the objective of examining the impact of ACEI/ARB therapy on the mortality of these patients.

## Methods

### Study design and population

**Fig. 1:**
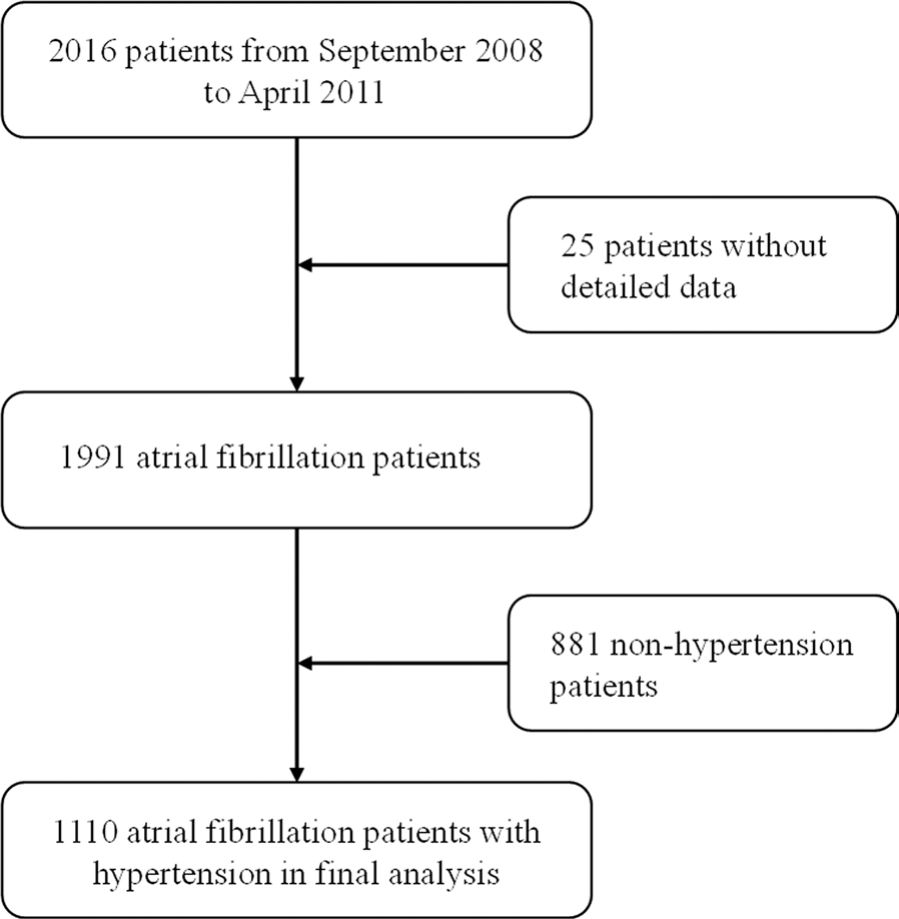
Flowchart of the study population

The Chinese AF registry study was designed as a multicenter prospective study. Between November 2008 and October 2011, 2016 consecutive AF patients from 20 emergency centers around China were enrolled (Fig. 1). Then, 25 subjects with incomplete data and 881 subjects without hypertension were excluded. Finally, 1110 patients with AF and hypertension were included in the final analysis, who were categorized into two groups based on whether they received ACEI/ARB therapy or not. The present study complied with the Declaration of Helsinki, and the study protocol was approved by the ethics committee of the 20 emergency centers. Written informed consent was obtained from all the participants.

### Baseline data collection

Baseline data, including demographics, vital signs at admission, comorbidities, and medications, were retrieved from the medical records or by interviewing the patients. AF was categorized as paroxysmal, persistent, or permanent based on the clinical practice guidelines of AF [13]. Data of comorbidities were collected at admission regarding history of myocardial infarction, coronary artery disease (CAD), congenital heart disease, diabetes mellitus, heart failure, rheumatic heart disease, left ventricular hypertrophy (LVH), stroke or transient ischemic attack (TIA), sleep apnea, smoking, left ventricular ejection fraction (LVEF) < 40%, dementia, hyperthyroidism, chronic obstructive pulmonary disease (COPD), valvular heart disease, and prior major bleeding and the diagnoses were based on physicians’ clinical medical records. The CHADS_2_ score was calculated based on the clinical guidelines [14]. In addition, we collected the details of medication history, including anticoagulants, antiplatelet agents, and antiarrhythmic agents.

### Follow-up and endpoints

All patients were followed up for 1 year ± 4 weeks by trained researchers through telephonic interviews or outpatient visits who were blinded to the medical conditions of the patients. All-cause death was the primary endpoint of this study. The second endpoints were cardiovascular death, stroke, major adverse events (MAEs), and recurrence of paroxysmal AF. MAEs were defined as a composite endpoint, including all-cause death, stroke, major bleeding, and non-central nervous system (CNS) embolism. Cardiovascular death included deaths caused by sudden death or arrhythmia death, heart failure, stroke, myocardial infarction, and pulmonary embolus, while cases of hemorrhages, cancer, trauma, respiratory failure, infection, or death by unknown cause represented non-cardiovascular death.

### Statistical analysis

This study used IBM SPSS software version 26.0 (SPSS Inc., Chicago, IL, USA) and R version 4.0.4 (R Foundation for Statistical Computing, Vienna, Australia) for conducting statistical analyses. Continuous variables are described as mean ± standard deviation or median with interquartile ranges. Categorical variables were described as numbers and percentages. *P* values of continuous variables were compared by using Student’s *t* test or Mann–Whitney *U* test based on their distributions checked by the Kolmogorov–Smirnov test, while categorical variables were compared using Pearson’s chi-square tests. We conducted univariable and multivariable Cox regression to compute the hazard ratios (HR) and 95% confidence intervals (CI) for all-cause mortality, cardiovascular mortality, stroke, and MAEs. Risk factors adjusted in the multivariable Cox regression models included age, body mass index (BMI), heart rate, heart failure, rheumatic heart disease, significant valvular disease, stroke or transient ischemic attack histories, dementia, COPD, diuretics, warfarin, and CHADS_2_ score ≥ 2. Each Cox regression model was checked by Schoenfeld residuals and smoothed plots, and all variables conformed to the proportional hazard assumption. Kaplan–Meier survival curves were constructed and examined by log-rank tests. Furthermore, we conducted univariable and multivariable Logistic regression analysis for recurrence of paroxysmal AF. Subgroup analyses of the relationship between ACEI/ARB treatment and all-cause mortality were also performed. All statistical analyses were two-tailed and *P* values < 0.05 were considered statistically significant.

## Results

### Baseline characteristics

Among the 1110 patients with AF and hypertension, 574 (51.7%) received ACEI/ARB therapy. The baseline characteristics of this study population were presented in Table 1. The initial BP in the two groups was not significantly different. Patients in the ACEI/ARB group tended to have higher BMI but lower heart rates (all *P* < 0.05) than patients without ACEI/ARB therapy. The proportion of permanent AF cases in the ACEI/ARB group was higher (*P* < 0.05). Moreover, patients receiving ACEI/ARB therapy were more likely to have myocardial infarction, CAD, and heart failure (all *P* < 0.05). The ACEI/ARB therapy group had a higher percentage of patients with a LVEF < 40% and a CHADS_2_ score ≥ 2 (all *P* < 0.05). Patients who received ACEI/ARB therapy tended to receive diuretics, β-blockers, dihydropyridine calcium channel blockers, digoxin, aspirin, statins, and amiodarone (all *P* < 0.05).

**Table 1:** Baseline characteristics of patients combined with AF and hypertension according to ACEI/ARB

	Total (n = 1110)	ACEI/ARB (n = 574)	no-ACEI/ARB (n = 536)	*P* value
Male (n[%])	492 [44.3%]	263 [45.8%]	229 [42.7%]	0.300
Age (y)	74(66–80)	73(66–79)	74(67–80)	0.053
BMI (Kg/m^2^)	23.8(22.0–26.0)	24.0(22.1–26.7)	23.6(21.9–25.7)	0.027
SBP (mmHg)	140(124–152)	140(126–154)	140(122–155)	0.100
DBP (mmHg)	80(71–90)	80(74–90)	80(70–90)	0.109
Heart rate (beat/min)	96(80–120)	96(79–118)	98(80–122)	0.023
Type of AF (n[%])				0.019
Paroxysmal	232 [20.9%]	103 [17.9%]	129 [24.1%]	
Persistent	353 [31.8%]	180 [31.4%]	173 [32.3%]	
Permanent	525 [47.3%]	291 [50.7%]	234 [43.7%]	
**Comorbidities**				
Myocardial infarction (n[%])	104 [9.4%]	70 [12.2%]	34 [6.3%]	0.001
Coronary artery disease (n[%])	607 [54.7%]	343 [59.8%]	264 [49.3%]	< 0.001
Congenital heart disease (n[%])	11 [1.0%]	4 [0.7%]	7 [1.3%]	0.306
Diabetes mellitus (n[%])	243 [21.9%]	110 [19.2%]	133 [24.8%]	0.286
Heart failure (n[%])	374 [33.7%]	234 [40.8%]	140 [26.1%]	< 0.001
Rheumatic heart disease (n[%])	77 [6.9%]	41 [7.1%]	36 [6.7%]	0.780
Left ventricular hypertrophy (n[%])	221 [19.9%]	129 [22.5%]	92 [17.2%]	0.027
Previous stroke or TIA (n[%])	279 [25.1%]	136 [23.7%]	143 [26.7%]	0.252
Sleep apnea (n[%])	46 [4.1%]	24 [4.2%]	22 [4.1%]	0.949
Smoking (n[%])	238 [21.4%]	133 [23.2%]	105 [19.6%]	0.146
LVEF < 40% (n[%])	207 [18.6%]	129 [22.5%]	78 [14.6%]	0.001
Dementia (n[%])	31 [2.8%]	10 [1.7%]	21 [3.9%]	0.028
COPD (n[%])	137 [12.3%]	71 [12.4%]	66 [12.3%]	0.977
Hyperthyroidism (n[%])	27 [2.4%]	19 [3.3%]	8 [1.5%]	0.050
Valvular heart disease (n[%])	90 [8.1%]	53 [9.2%]	37 [6.9%]	0.155
Prior major bleeding (n[%])	25 [2.3%]	11 [1.9%]	14 [2.6%]	0.435
CHADS_2_ score ≥ 2 (n[%])	846 [76.2%]	454 [79.1%]	392 [73.1%]	0.020
**Medication status**				
Diuretic (n[%])	422 [38.0%]	282 [49.1%]	140 [26.1%]	< 0.001
β blocker (n[%])	567 [51.1%]	311 [54.2%]	256 [47.8%]	0.033
Calcium channel blocker (n[%])	412 [37.1%]	202 [35.2%]	210 [39.2%]	0.169
Digoxin (n[%])	278 [25.0%]	190 [33.1%]	88 [16.4%]	< 0.001
Aspirin (n[%])	680 [61.3%]	405 [70.6%]	275 [51.3%]	< 0.001
Clopidogrel (n[%])	98 [8.8%]	55 [9.6%]	43 [8.0%]	0.360
Statin (n[%])	363 [32.7%]	253 [44.1%]	110 [20.5%]	< 0.001
Oral anticoagulants (n[%])	147 [13.2%]	79 [13.8%]	68 [12.7%]	0.597
Amiodarone (n[%])	121 [10.9%]	74 [12.9%]	47 [8.8%]	0.028
Propafenone (n[%])	37 [3.3%]	18 [3.1%]	1 [3.5%]	0.705

ACEI: angiotensin-conver ting enzyme inhibitor; AF: atrial fibrillation; ARB: angiotensin II receptor blocker; BMI: Body mass index values; CCB: calcium channel blocker; COPD: chronic obstructive pulmonary disease; DBP: diastolic blood pressure; LVEF: left ventricular ejection fraction; TIA: transient ischemic attack; SBP systolic blood pressure

### Outcomes of 1-year follow-up

As shown in Table 2, over a 1-year ± 4 weeks follow-up, 169 all-cause deaths (15.2%), 100 cardiovascular deaths (9.0%), and 255 MAEs (23.0%) occurred, while 98 patients suffered from stroke (8.8%) in this study. Among the 232 paroxysmal AF patients, the ACEI/ARB and non-ACEI/ARB groups had 22 and 21 cases of recurrences (21.4% vs. 16.3%, *P* = 0.32), respectively (Additional file 1: Table S2). The all-cause mortality was 12.5% and 18.1% in patients receiving ACEI/ARB therapy versus those not receiving ACEI/ARBs, respectively (*P* = 0.010). Patients receiving ACEI/ARB therapy had a lower incidence of MAEs (19.5% vs. 24.8%, *P* = 0.005) than those not receiving ACEIs/ARBs. However, the risk of cardiovascular death (8.4% vs. 9.7%, *P* = 0.436) and stroke (8.0% vs. 9.7%, *P* = 0.322) was comparable between both groups.

**Table 2:** Association between ACEI/ARB therapy and one-year outcomes in AF patients with hypertension

Outcomes	Total	ACEI/ARB	no ACEI/ARB	*P* value	Univariable analysis		Multivariable analysis*	
	(n = 1110)	(n = 574)	(n = 536)		HR (95%CI)	*P* value	HR (95% CI)	*P* value
All-cause death	169 [15.2%]	72 [12.5%]	97 [18.1%]	0.010	0.658 (0.485–0.893)	0.007	0.605 (0.431–0.849)	0.004
Cardiovascular death	100 [9.0%]	48 [8.4%]	52 [9.7%]	0.436	0.778 (0.521–1.162)	0.220	0.585 (0.372–0.921)	0.020
Stroke	98 [8.8%]	46 [8.0%]	52 [9.7%]	0.322	3.391 (0.353–32.602)	0.290	0.721 (0.468–1.111)	0.138
MAEs	255 [23.0%]	112 [19.5%]	143 [24.8%]	0.005	0.692 (0.540–0.886)	0.004	0.651 (0.496–0.855)	0.002

^*^Adjusted for age, body mass index, heart rate, heart failure, significant valvular disease, rheumatic heart disease, prior stroke/transient ischemic attack, dementia, chronic obstructive pulmonary disease, diuretic, warfarin, CHADS_2_ score ≥ 2, statins, beta-blockers, aspirin, digoxin, and amiodarone

**Fig. 2:**
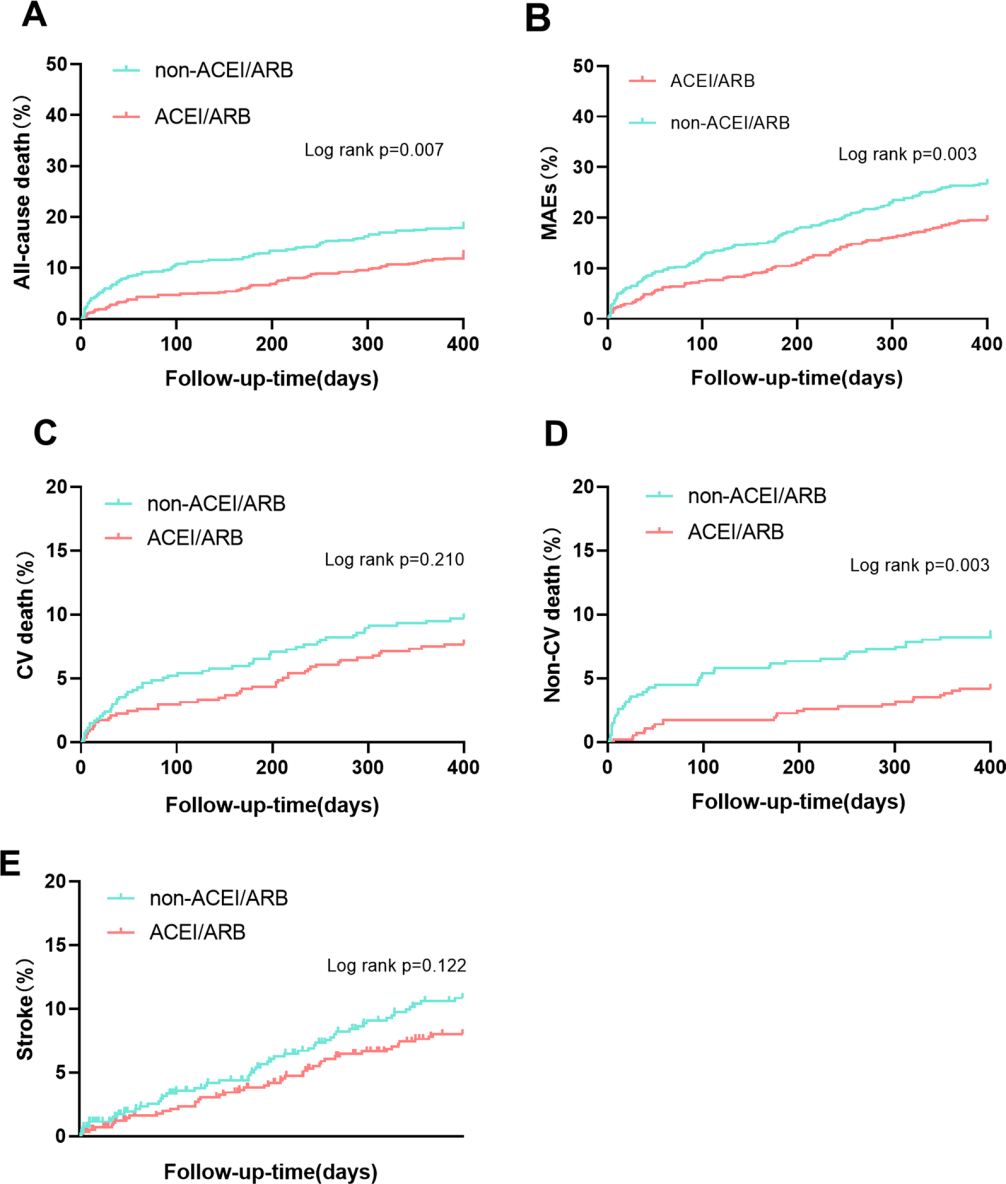
The Kaplan–Meier analysis according to whether receive ACEI/ARB therapy. **A** All-cause mortality; **B** MAEs; **C** CV death; **D** Non-CV death; **E** Stroke. ACEI: angiotensin-converting enzyme inhibitor; ARB: angiotensin II receptor blocker; CV: cardiovascular; MAEs: major adverse events

**Fig. 3:**
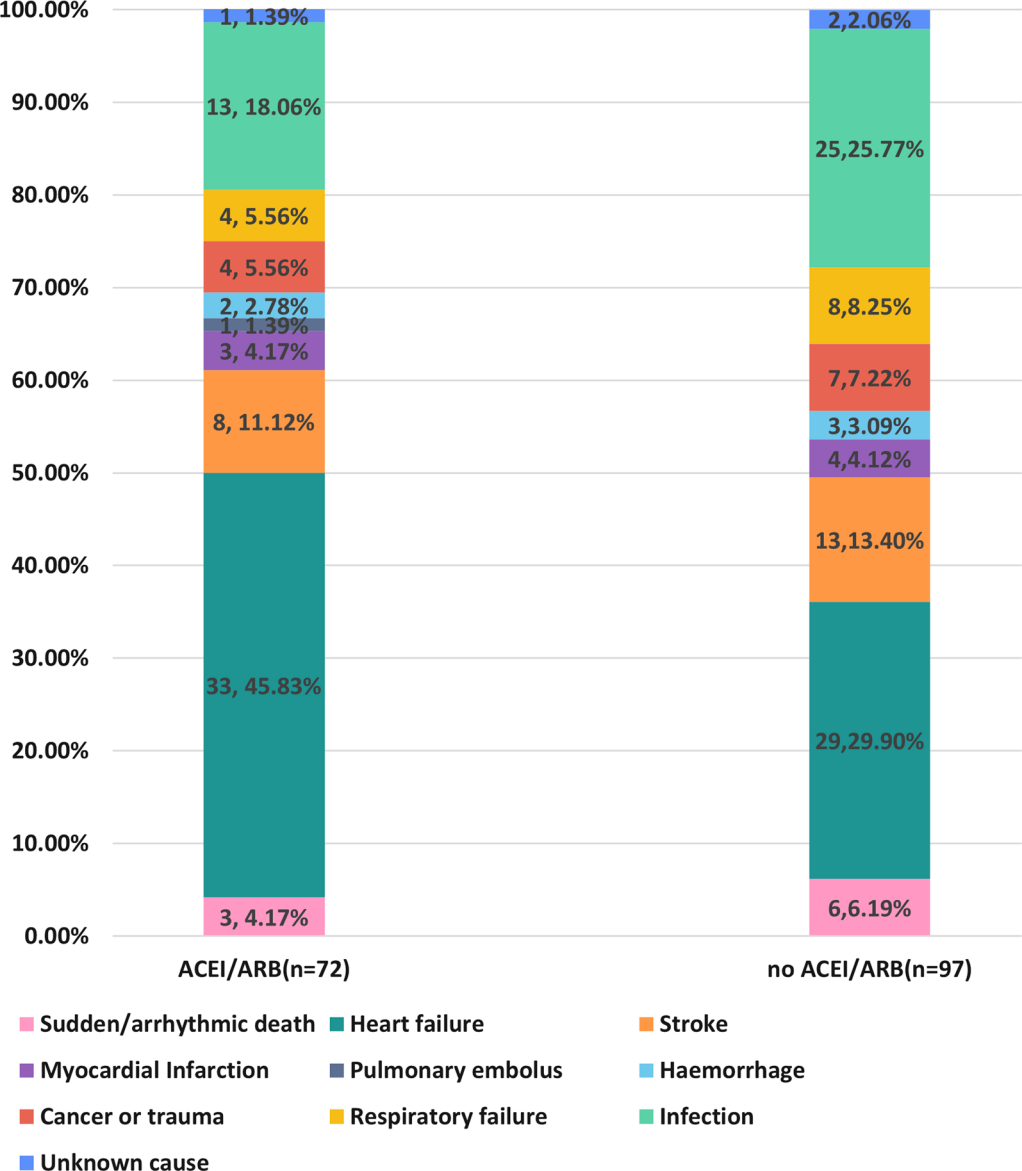
The specific reason for 1-year death in AF patients with hypertension according to ACEI/ARB

Kaplan–Meier survival analysis (Fig. 2) suggested that the ACEI/ARB group had a significantly lower risk of all-cause death (*P* = 0.007) and MAEs (*P* = 0.003). Cardiovascular death and stroke rates were lower in patients who received ACEI/ARB therapy, but the difference between both groups was not significant. The risk of non-cardiovascular death in patients receiving ACEI/ARB therapy was significantly lower than that in the non-ACEI/ARB group (*P* = 0.003). Figure 3 shows the specific reason for death, heart failure being the main cause, followed by infection in our emergency department (ED) patients with AF and hypertension. Interestingly, the ACEI/ARB group was associated with a significantly lower rate of infection-induced deaths (*P* < 0.05) (Additional file 1: Table S1).

Univariate Cox regression analysis (Table 2) revealed that ACEI/ARB use was significantly associated with a reduced risk of all-cause mortality (HR 0.658, 95% CI 0.485–0.893, *P* = 0.007) and MAEs (HR 0.692, 95% CI 0.540–0.886, *P* = 0.004) in our cohort. While ACEI/ARB therapy reduced the risk of cardiovascular mortality (HR 0.778, 95% CI 0.521–1.162, *P* = 0.220) and stroke (HR 3.39, 95% CI 0.353–32.602, *P* = 0.290), the effect was non-significant. The potential confounders included age, BMI, heart rate, history of heart failure, significant valvular disease, rheumatic heart disease, stroke or TIA, dementia, COPD, diuretic, warfarin, CHADS_2_ score ≥ 2, statins, beta-blockers, aspirin, digoxin, and amiodarone. After adjusting for these factors, the multivariate Cox regression analysis indicated that ACEI/ARB was still independently associated with a reduced risk of all-cause mortality (HR 0.605, 95% CI 0.431–0.849, *P* = 0.004). Moreover, ACEI/ARB therapy was significantly associated with a reduced risk of cardiovascular mortality (HR 0.585, 95% CI 0.372–0.921, *P* = 0.020) and MAEs (HR 0.651, 95% CI 0.496–0.855, *P* = 0.002) after adjustments for confounders, while ACEI/ARB had a neutral effect on stroke (HR, 0.721; 95% CI 0.468–1.111; P = 0.138) (Table 2). In addition, both the univariate (OR, 1.40; 95% CI 0.72–2.71) and multivariate (OR, 1.12; 95% CI 0.47–2.64) Logistic regression indicated that no significant association between the use of ACEI/ARB and preventing the recurrence of paroxysmal AF (all* P* > 0.05). (Additional file 1: Table S2).

**Fig. 4:**
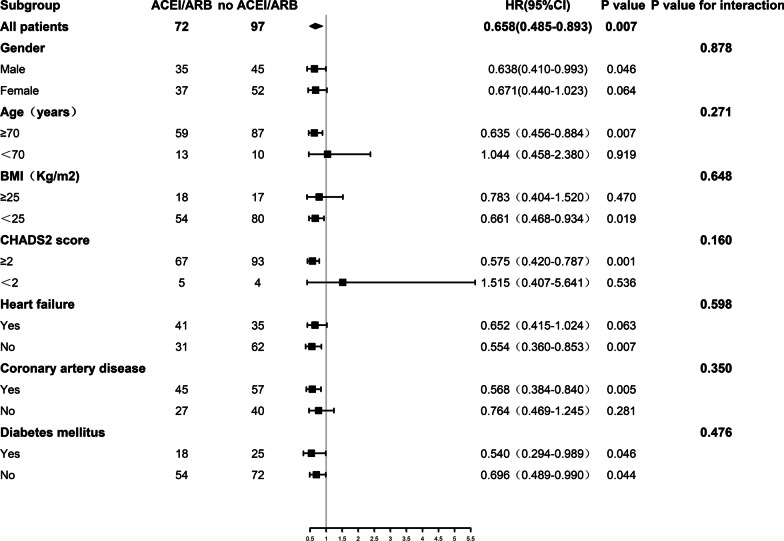
Subgroup analysis for associations between ACEI/ARB therapy and one-year all-cause mortality in patients with AF and hypertension

Patients receiving ACEI/ARB consistently showed a lower risk of all-cause mortality than those not receiving RAAS inhibitors, regardless of sex, age, CHADS_2_ score, heart failure, CAD, and diabetes mellitus. Moreover, there were no significant interactions between the subgroups and all-cause mortality (Fig. 4).

## Discussion

The results of this cohort study showed that ED patients with AF and hypertension who received ACEI/ARB therapy had a lower incidence of all-cause death (12.5% vs. 18.1%, *P* = 0.010) and MAEs (19.5% vs. 24.8%, *P* = 0.005) than patients who did not receive ACEI/ARBs. In particular, after adjusting for the potential confounders, including drugs that are effective in preventing the risk of cardiovascular adverse events such as statins, beta-blockers, aspirin, digoxin, amiodarone, and other comorbidities, the multivariable Cox regression analysis results showed that ACEI/ARB therapy was independently associated with a reduced risk of all-cause mortality, cardiovascular mortality, and MAEs. In addition, the subgroup analysis results were consistent with the overall results.

Generally, it is difficult to determine if ACEI/ARB brought the benefit influence beyond the effects of blood-pressure lowering. Thus, we selected AF patients having hypertension as the study population initially and followed the example of the Denmark study [11], which considered the risk of stroke as a sign of BP lowering. Hypertension is recognized as the single strongest effector for stroke [15], the risk of stroke is mainly affected by controlling BP rather than RAAS inhibition. When the BP is controlled within a certain range, the reduction in stroke incidence is similar, which is irrelevant to the type of antihypertensive drugs [15]. The initial BP values of the two groups were comparable in our study. Eventually, the incidence of stroke was not significantly different between both groups; however, the ACEI/ARB group had a reduced risk of all-cause mortality, cardiovascular deaths, and MAEs. These findings indicated inhibiting RAAS might contribute to the improvement of prognosis in patients with AF and hypertension independent of the effect of blood pressure controlling.

ACEI/ARB has been proven as a significant influencer in the primary prevention of AF and became an important part of upstream therapy [11]. In addition, the effect of ACEI/ARB in secondary prevention of AF was widely investigated, but the results were controversial. The results of the EAST-AFNET study suggested that early rhythm-control therapy by anti-arrhythmia drugs and catheter ablation reduced cardiovascular risk significantly [22]. Rhythm control as a part of better symptom control becomes a pivotal topic in the treatment of AF patients [13]. However, several randomized control trials (GISSI-AF, ACTIVE-I, ANTIPAF, and TOPCAT) indicated that RAAS inhibitors (Valsartan, Irbesartan, Olmesartan, and Spironolactone) were ineffective in maintaining sinus rhythm and preventing recurrence of AF [23–26]; the results of these trials were consistent with our study (Additional file 1: Table S2). We speculated the beneficial effect of ACEI/ARB on the prognosis of AF patients with hypertension might be beyond the anti-arrhythmic effect and was probably not associated with maintenance of sinus rhythm control. In fact, limited studies have evaluated the relationship between the use of ACEI/ARB and the mortality of AF. The LIFE study revealed that losartan was associated with a lower risk of cardiovascular death and stroke in AF patients with hypertension and LVH [27]. Similar to the results of the LIFE study, our findings suggested that ACEI/ARB therapy was independently associated with a reduced risk of cardiovascular mortality and MAEs in AF, not limited to LVH. In contrast, in a registry study by Fernandez et al., which included 9365 AF patients aged ≥ 75 years, ACEI/ARB therapy did not reduce the rates of all-cause deaths and cardiovascular events [28]. The investigators of that study interpreted their results to be limited to the older patients with AF. Whereas, our study included patients of all ages with AF and hypertension and in our subgroup analysis conducted to evaluate for the impact of age on the mortality of these patients, the overall results were not affected by old age.

Notably, the results of the univariate Cox regression analysis in our study showed no significant difference in cardiovascular mortality between both groups (Table 2). Other medications which were proved to be effective to reduce cardiovascular risks such as statins, beta-blockers, aspirins and comorbidities might influence the impact of ACEI/ARB on cardiovascular deaths. However, after adjusting for these variables, patients treated with ACEI/ARB were independently associated with a reduced risk of cardiovascular death (*P* < 0.05). Interestingly, as shown in Fig. 2D, the risk of non-cardiovascular death was significantly lower in patients treated with ACEI/ARB than that in patients without ACEI/ARB therapy (*P* < 0.05), which was attributed to the significantly lower rates of infection-induced deaths in the ACEI/ARB group (18.06% vs. 25.77%, *P* < 0.05) (Fig. 3). According to our findings and previous studies [29, 30], infection-induced death needs more attention in clinical research, since it is among the main leading causes of death in AF patients. One retrospective study including 52 727 sepsis patients showed that ACEI/ARB therapy was associated with reduced short-term mortality caused by sepsis, when compared with patients not receiving ACEI/ARB therapy [31]. Even the impact of ACEI/ARB on Corona Virus Disease 2019 (COVID-19) progression has been a hot topic during the COVID-19 epidemic [32]. According to an observational study in patients with hypertension and COVID-19, ACEI/ARB therapy was associated with a lower risk of mortality [33]. The mechanism underlying the protective role of ACEI/ARB in sepsis and other infectious diseases may be related to RAAS inhibition [34]. As the primary effector of the RAAS, angiotensin II induces upregulation of pro-inflammatory cytokines—tumor necrosis factor-α and interleukin-1 [35]. Moreover, in animal models of sepsis, ACE/ARB treatment was associated with the reduction of pro-inflammatory cytokine activity and improvement in survival rates [35]. We speculated that the protective effect brought by ACEI/ARBs on the survival of AF patients was partly attributable to the reduction of pro-inflammatory cytokine activity because of RAAS inhibition. However, this hypothesis requires further research in the future.

In this cohort, the all-cause mortality of AF patients with hypertension was 15.2% per year and the cardiovascular mortality rate was 9.0% per year, which accounted for 59.17% of all-cause mortality in this cohort. The RELY study reported an all-cause mortality of 11% for AF [16]. In contrast, the GARFIELD-AF study [17] reported a mortality rate of 3.83% per year for AF, in which 60.8% of the AF patients received anticoagulant therapy. The higher mortality in our study can be explained by only 13.2% of the patients administered to anticoagulant therapy corresponding to 76.2% of patients with CHADS_2_ ≥ 2 in the study population, since the 20 centers included were representing different levels of medical care centers from urban to rural areas around China. Another possible reason was that only ED patients were included in our study population who were frailer and seemed to have other debilitating conditions. In 2017, Atrial fibrillation Better Care Pathway (ABC pathway) was suggested to simplify and standardize the care process of AF [36]. The 2020 European Society of Cardiology (ESC) guidelines recommended the ABC pathway to manage AF patients [13]. A meta-analysis on 285 000 AF patients revealed that adherence to ABC pathway was related to a reduced risk of all-cause mortality and CV deaths [37]. Although BP control is an important treatment in the management of AF patients with hypertension, oral anticoagulation (A-Anticoagulation/Avoid stroke) should be initiated without delay after appropriate assessment by the CHA_2_DS_2_-VASc score, especially for those with critical illness. Moreover, treating hypertension to reduce the overall cardiovascular risk remains a part of the ABC pathway (C – Comorbidities). The ESC guidelines highlighted that strict BP control with anticoagulation therapy is important for reducing the overall cardiovascular risk [13]. Thus, our results support the ESC guidelines for managing AF, adhering to the ABC pathway is fundamental to reducing mortality for AF patients [13, 36].

### Limitations

The present study had some potential limitations. First, as a multicenter study, systematic errors and biases might affect the accuracy of the final results. Second, the study was limited to the short follow-up duration of 1-year and had a relatively small sample size. Therefore, larger randomized clinical trials are necessary to verify these findings. Third, we did not collect some clinical parameters such as echocardiographic or global longitudinal strain measurements; hence, the changes in cardiac remodeling that may have occurred during the period of ACEI/ARB treatment could not be evaluated. Future research is needed to verify the association between RAAS-inhibitor therapy and prevent atrial remodeling. Besides, it would be more instructive if data on the duration of ACEI/ARB therapy is available.

Despite these limitations, our study provides evidence for ACEI/ARB to improve the survival of AF patients with hypertension and brings new light on choosing antihypertensive drugs for them. Besides, our results support the ESC guidelines and ABC pathway to better manage AF patients.

## Conclusion

In this study, our main finding was that ACEI/ARB therapy was associated with a reduced risk of all-cause mortality, cardiovascular mortality, and MAEs in ED patients with AF and hypertension. These findings indicate that, in addition to blood pressure control therapy, preventing the activation of the RAAS system might improve prognosis in patients with AF and hypertension. These results provide evidence for a tertiary preventive treatment for patients with atrial fibrillation and hypertension.

## Supplementary Information

**Additional file 1:** Supplementary material. **Table S1:** Causes of deaths in AF patients with hypertension. **Table S2:** Associations between ACEI/ARB and recurrence in paroxysmal AF with hypertension.

## Data Availability

The datasets generated and analyzed during the current study are not publicly available due privacy and ethical restrictions but are available from the corresponding author on reasonable request.

## Abbreviations

ABC pathway: Atrial fibrillation Better Care Pathway
ACEI: Angiotensin-converting enzyme inhibitor
AF: Atrial fibrillation
ARB: Angiotensin II receptor blocker
BMI: Body mass index values
BP: Blood pressure
CAD: Coronary artery disease
CCB: Calcium channel blocker
CI: Confidence interval
COPD: Chronic obstructive pulmonary disease
COVID-19: Corona Virus Disease 2019
CV: Cardiovascular
CNS: Central nervous system
DBP: Diastolic blood pressure
ED: Emergency department
ESC: European Society of Cardiology
HR: Hazard ratio
LVEF: Left ventricular ejection fraction
LVH: Left ventricular hypertrophy
MAEs: Major adverse events
RAAS: Renin–angiotensin–aldosterone system
TIA: Transient ischemic attack
SBP: Systolic blood pressure
