# Supplementary material for: Impact of renin–angiotensin–aldosterone-system inhibitor drugs on mortality in patients with atrial fibrillation and hypertension
Source: BMC Cardiovasc Disord. 2022 Apr 1;22:141. doi: 10.1186/s12872-022-02580-2 (PMC8973677; doi:10.1186/s12872-022-02580-2)
Supplement: Supplementary file 1 — Additional file 1: Supplementary material. Table S1: Causes of deaths in AF patients with hypertension. Table S2: Associations between ACEI/ARB and recurrence in paroxysmal AF with hypertension. [file 12872_2022_2580_MOESM1_ESM.docx]

**Supplementary material**

**Table S1**

**Causes of deaths in AF patients with hypertension**

| **Causes of deaths** | **Total** | **ACEI/ARB** | **No ACEI/ARB** | **P-value** |
| --- | --- | --- | --- | --- |
|  | **(n=169)** | **(n=72)** | **(n=97)** |  |
| **Cardiovascular deaths** | 100[59.17%] | 48[66.67%] | 52[53.61%] | 0.436 |
| Sudden/arrhythmic death | 9[5.32%] | 3[4.17%] | 6[6.19%] | 0.268 |
| Heart failure | 62[36.69%] | 33[45.83%] | 29[29.90%] | 0.806 |
| Stroke | 21[12.43%] | 8[11.12%] | 13[13.40%] | 0.207 |
| Myocardial Infarction | 7[4.14%] | 3[4.17%] | 4[4.12%] | 0.638 |
| Pulmonary embolus | 1[0.59%] | 1[1.39%] | 0 | 0.334 |
| **Non-cardiovascular deaths** | 69[40.83%] | 24[33.33%] | 45[46.39%] | 0.004 |
| Hemorrhage | 5[2.96%] | 2[2.78%] | 3[3.09%] | 0.599 |
| Cancer or trauma | 11[6.51% ] | 4[5.56%] | 7[7.22%] | 0.306 |
| Respiratory failure | 12[7.10%] | 4[5.56%] | 8[8.25%] | 0.200 |
| Infection | 38[22.48%] | 13[18.06%] | 25[25.77%] | 0.028 |
| Unknown cause | 3[1.78%] | 1[1.39%] | 2[2.06%] | 0.524 |

**Table S2**

**Associations between ACEI/ARB and recurrence in paroxysmal AF with hypertension**

|  | **ACEI/ARB**  (**n=103**) | **No ACEI/ARB**  (**n=129**) | **P-value** |
| --- | --- | --- | --- |
| **Recurrence** | 22 (21.4%) | 21 (16.3%) | 0.32 |
|  | **OR** | **95% CI** | **P-value** |
| **Univariable analysis** | 1.40 | 0.72-2.71 | 0.32 |
| **Multivariable analysis*** | 1.12 | 0.47-2.64 | 0.80 |

*Adjusted for age, sex, heart rate, BMI, systolic blood pressure, diastolic blood pressure, coronary artery disease, heart failure, stroke/TIA history, smoking, COPD, dihydropyridine calcium channel blockers, non - dihydropyridine calcium channel blockers, beta-blockers, digoxin, statins, antiplatelet agents, amiodarone.
